# Morphometric Analysis of Recognized Genes for Autism Spectrum Disorders and Obesity in Relationship to the Distribution of Protein-Coding Genes on Human Chromosomes

**DOI:** 10.3390/ijms17050673

**Published:** 2016-05-05

**Authors:** Austen B. McGuire, Syed K. Rafi, Ann M. Manzardo, Merlin G. Butler

**Affiliations:** Departments of Psychiatry & Behavioral Sciences and Pediatrics, University of Kansas Medical Center, Kansas City, KS 66160, USA; austenmcguire@gmail.com (A.B.M.); rafigene@yahoo.com (S.K.R.); amanzardo@kumc.edu (A.M.M.)

**Keywords:** G-negative euchromatin, G-positive heterochromatin, chromosome organization, high-resolution chromosome ideograms, protein-coding genes, obesity genes, autism spectrum disorder (ASD) genes

## Abstract

Mammalian chromosomes are comprised of complex chromatin architecture with the specific assembly and configuration of each chromosome influencing gene expression and function in yet undefined ways by varying degrees of heterochromatinization that result in Giemsa (G) negative euchromatic (light) bands and G-positive heterochromatic (dark) bands. We carried out morphometric measurements of high-resolution chromosome ideograms for the first time to characterize the total euchromatic and heterochromatic chromosome band length, distribution and localization of 20,145 known protein-coding genes, 790 recognized autism spectrum disorder (ASD) genes and 365 obesity genes. The individual lengths of G-negative euchromatin and G-positive heterochromatin chromosome bands were measured in millimeters and recorded from scaled and stacked digital images of 850-band high-resolution ideograms supplied by the International Society of Chromosome Nomenclature (ISCN) 2013. Our overall measurements followed established banding patterns based on chromosome size. G-negative euchromatic band regions contained 60% of protein-coding genes while the remaining 40% were distributed across the four heterochromatic dark band sub-types. ASD genes were disproportionately overrepresented in the darker heterochromatic sub-bands, while the obesity gene distribution pattern did not significantly differ from protein-coding genes. Our study supports recent trends implicating genes located in heterochromatin regions playing a role in biological processes including neurodevelopment and function, specifically genes associated with ASD.

## 1. Introduction

Over the course of evolution, the architecture of chromosome structure has become substantially complex with the specific assembly and configuration of each chromosome influencing gene expression and function [1]. There is a need within the field of genetics to better understand chromosome structure and organization, including factors that influence chromosome function and gene location [2]. One aspect of chromosomal organizational research has sought to understand chromatin architecture, the combination of DNA and proteins within a nucleosome with condensation of the expansively long strands of DNA to help store and maintain DNA, the building blocks of genes (e.g., [3,4]). The chromatin of eukaryotic chromosomes is divided into two main categories: euchromatin and heterochromatin. These regions in each chromosome contain different histone modifications that impact gene expression and DNA packaging styles [5].

Euchromatin chromosome regions tend to be less compact than the more tightly packed heterochromatin regions [5]. At most times during the cell cycle, the euchromatin region decondenses during interphase [6], and the less condensed packaging style is associated with greater gene transcription and activity with early replication [7,8]. Unlike euchromatin, heterochromatin regions are thought to contain less transcription and gene activity with later replication. Heterochromatin is further divided into two subtypes with distinct gene activity (expression) profiles: constitutive and facultative [9,10,11]. Constitutive heterochromatin is thought to be inactive (not expressed) while facultative heterochromatin may be either active or inactive [12] and less studied compared to the euchromatin regions in the genome [5,13,14]. The remodeling of facultative heterochromatin is known to silence euchromatin-based functional genes due to the gradual gain of lysine methylation in heterochromatic regions, but DNA sequencing of the heterochromatin regions is challenging due to the length and repetitive nature of the code [14]. Recent evidence suggests that heterochromatin may play an important role in development [13,15,16], such as the role of facultative heterochromatin in transcription-associated chromatin remodeling complexes. Heterochromatin regions were once thought to be largely inactive and only important in gene-expression silencing, but facultative heterochromatin is known to change and decondense, thereby allowing for transcription [10,17]. Chromatin remodeling is therefore reversible and dynamic but required for timely activation of functional genes during development [13]. More research is needed to better understand and elucidate differences and similarities between euchromatin and heterochromatin regions and their influence on gene position and activity.

Chromosome-banding methods have been used to visually delineate euchromatin from heterochromatin regions in the study of human chromosomes for more than 45 years. Chromosome banding is applied in the clinical setting for identification of structural and numerical anomalies with an average band accounting for about five megabases of DNA. Chromosome-banding techniques assimilate the DNA nucleotide sequence, associated proteins and functional organization of the chromosome through the use of DNA-staining procedures, most commonly Giesma stain. Giemsa preferentially stains transcriptionally less active, AT-nucleotide-rich sequences associated with heterochromatin regions, referred to as G-dark or Giemsa-positive, while the relatively active GC-nucleotide-rich euchromatin regions stain less intensely, leading these locations to be referred to as G-light or Giemsa-negative [18]. Earlier studies report that GC-nucleotide-poor regions constitute 63% of the human genome, whereas GC-nucleotide-rich regions make up 37% [19].

Protein-coding genes are responsible for the production of proteins to support cellular growth and functioning. The number of protein-coding genes has been estimated at 21,000 (e.g., [14]) and are distributed unevenly among the 24 different chromosomes as represented on high-resolution Ensembl ideograms (Ensembl, available at: http://uswest.ensembl.org/Homo_sapiens/Location/Genome?redirect=no) [20]. The greatest number of protein-coding genes are located on chromosomes 1, 19, 11, and 17, respectively, with the least number of protein-coding genes located on chromosomes Y, 21, 13, and 18 [21], not reflecting the size of the chromosome. However, chromosome 1 is considered the largest chromosome, and chromosome 19 is one of the smallest [21]. The distribution of genome-wide protein-coding genes among the chromosomes is reported by numerous credited sources, such as Ensembl [21] and the Genetics Home Reference provided through the National Institutes of Health (available at: http://ghr.nlm.nih.gov/chromosomes); however, less is known about the distribution of protein-coding genes among euchromatin and heterochromatin regions of the chromosomes. Distribution patterns associated with chromosome banding and proximity to fragile sites (regions susceptible to changes, disturbances and instability) could affect gene function or their location in relationship to subsets of protein-coding genes impacting different organ systems’ development and function.

The current study was set forth to descriptively characterize and advance knowledge on the distribution of known genome-wide protein-coding genes in relationship to the G-negative euchromatin, G-positive heterochromatin banding regions and fragile sites, chromosome band locations and chromosome size and disease states for genes recognized in autism spectrum disorders (ASD) and obesity [22,23]. Analyzing the distribution and relationship of genome-wide protein-coding genes in comparison with clinically relevant and associated genes for neurodevelopment and brain function (e.g., ASD) and those involved in the peripheral system (e.g., obesity) could increase our understanding of gene location and function influenced by chromosomal euchromatin and heterochromatin regions in development and disease. Genome-wide protein-coding genes associated with these two gene disorder groups will encompass the full range of the human genome with a large number of genes distributed across the 24 chromosomes. ASD is a spectrum of neurological disorders with known genetic influences and estimated heritability as high as 90% [24], while obesity is a systemic-based energy imbalanced disorder with an average heritability of approximately 50% [25]. The number of recognized genes and their location in both disease state gene sets have recently been summarized [26,27]. The functional status of known and candidate ASD and obesity genes could be implied by their locations within the predominately active G-negative euchromatin or inactive G-positive heterochromatin bands on high-resolution chromosome ideograms. Hence, the purpose of the current study is to compile and examine the distribution of all known genome-wide protein-coding genes from published ideograms among the individual human chromosomes and their location in euchromatin and heterochromatin regions at each band level. Additionally, we will compare for the first time the distribution of protein-coding genes with clinically relevant and known ASD and obesity disease-causing genes to characterize and compare their relationship at the chromosome level and to recognize any deviation from the total protein-coding gene distribution patterns.

## 2. Results and Discussion

**Figure 1:**
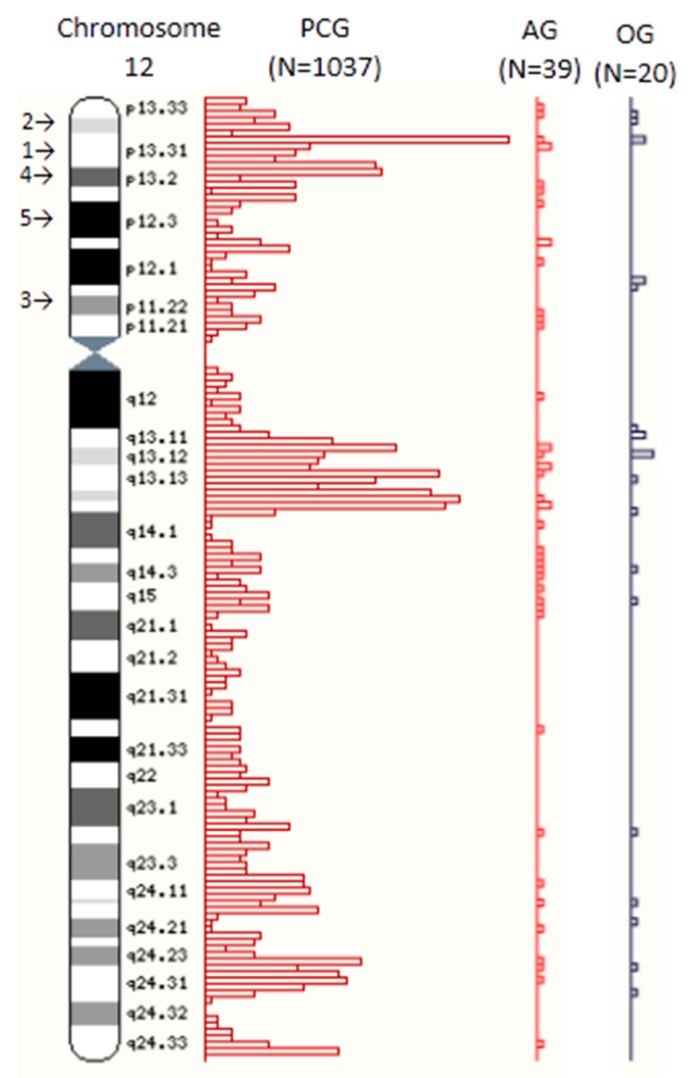
Sample chromosome ideogram with protein-coding, autism and obesity gene frequency distributions by Giemsa band. Ideogram representation of chromosome 12 taken from Genome Reference Consortium Ensembl website (http://uswest.ensembl.org/Homo_sapiens/Location/Genome) [21]. PCG = Protein-coding gene distribution, AG = Autism gene distribution, OG = Obesity gene distribution. 1 = Example of color 1 (euchromatin), 2 = Example of color 2 (heterochromatin), 3 = Example of color 3 (heterochromatin), 4 = Example of color 4 (heterochromatin), 5 = Example of color 5 (heterochromatin).

**Figure 2:**
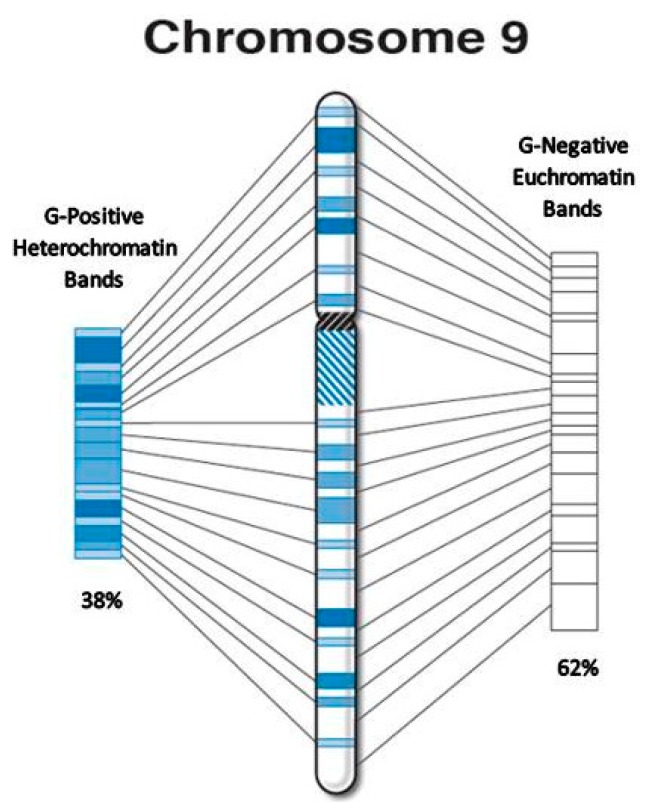
Sample stacked chromosome ideogram.

We conducted a morphometric analysis of published high-resolution chromosome ideograms to descriptively characterize the distribution and location of currently recognized relevant candidate or known genes for ASD, obesity and the total number of genome-wide protein-coding genes (see Figure 1). We measured and recorded the physical size (length in millimeters) of each chromosome as rendered ideogram representation of metaphase chromosomes along with their gene group status (*i.e.*, protein-coding [20], ASD [27], or obesity [26]). The location for each gene and distribution were determined from data collected from authoritative websites or published peer-reviewed sources. We then determined the location of these genes either on G-negative euchromatin bands or G-positive heterochromatin bands (and sub-bands) across each chromosome-based ideogram (see Figure 2). We determined their regional and chromosomal distribution patterns, and studied their position in relation to the physical size of the G-negative and G-positive chromatin as seen at the 850 high-resolution band level on the ISCN (2013) chromosome ideograms developed and scaled from cytological data [18] which are mostly equivalent to the high-resolution Ensembl ideograms, or in relation to the known genome-wide protein-coding, ASD, and obesity gene locations across the chromosomes. The Ensembl ideograms are based on chromosomes in the uncondensed state and may not necessarily reflect differential chromatin condensation in the structure of a metaphase chromosome whereby the heterochromatin may occupy different linear space than the euchromatin. However, a large positive correlation was found between the number of protein-coding and ASD genes (*r* = 0.65) per chromosome and between protein-coding and obesity genes (*r* = 0.85) per chromosome.

### 2.1. Gene Distributions

**Figure 3:**
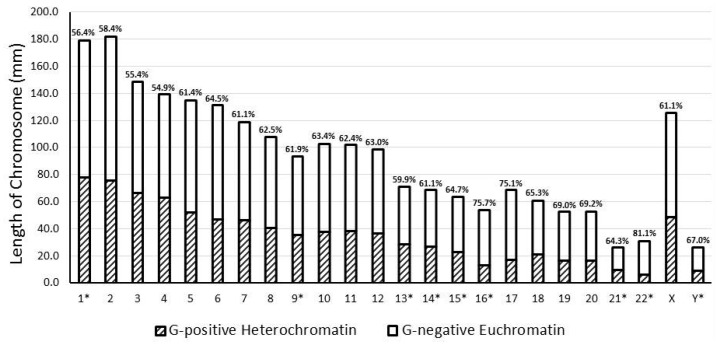
Giemsa band distributions as a proportion of chromosome length. * = Length of qh, centromeric and/or acrocentric chromosome p arm regions were excluded. Percentage above bar for each chromosome represents the proportion of G-negative euchromatin per chromosome.

Our overall measurements followed known and established banding patterns based upon chromosome size (see Table 1 and Figure 3). Chromosomes were arranged in size from number 1 to chromosome Y, except for the X chromosome and when the removal of the qh, centromeric, and acrocentric chromosome p arm regions altered this pattern. For example, chromosome 1 is the longest chromosome, but after removal of the qh region, it became shorter in size than chromosome 2. Altogether, G-negative euchromatin regions encompassed 56.4% of the genome (see Table 1), which contrasts earlier reports of GC-nucleotide-rich regions which only constitute approximately 37% of the human genome [19]. Each chromosome appears to follow this same distribution of approximately 60% for G-negative euchromatin DNA and 40% for G-positive heterochromatin DNA. However, chromosomes 16, 17 and 22 deviated from this pattern with >70% G-negative euchromatin DNA. These chromosomes had at least a threefold difference in euchromatin *vs*. heterochromatin DNA with chromosome 22 having the highest G-negative euchromatin/G-positive heterochromatin ratio of 4.28. This observation deviated considerably from G-negative euchromatin/G-positive heterochromatin expected ratios based on similarly sized chromosomes (*i.e.*, chromosome 21 (1.80 ratio) and chromosome Y (2.03 ratio)).

Protein-coding, ASD, and obesity genes followed a similar distributional pattern across the genome (all 24 chromosomes) based upon the relative length of the individual chromosome (see Table 2) with the number of genes located on a chromosome proportional to the length of the chromosome. As chromosome size decreases, the percentage of total genes per chromosome also decreases. For example, chromosome 1 is the largest chromosome, prior to removal of the qh region, and harbors one of the greatest numbers of protein-coding, ASD, and obesity genes, whereas chromosome 22 is one of the smallest chromosomes and contains one of the smallest numbers of protein-coding, ASD, and obesity genes. Deviations from normal patterns were found, e.g., for chromosome 19 which encompassed 2.3% of the total genome size but contained 7.2% of the total number of protein-coding genes. Chromosome 11 makes up 4.6% of the genome length but possessed 7.4% of obesity genes. Chromosome X makes up 5.6% of the genome length but contained 9.1% of the ASD genes reflecting the established gender disparity with male preponderance seen in ASD (4:1 male:female) [28]. The influence of the observed deviations relative to the typical distribution pattern is unclear and will require further research.

This review of morphometric and Giemsa banding chromosome characteristics with respect to the distribution of selected gene groups per length of chromosome did find that many chromosomes possessed a higher proportion of protein-coding genes (e.g., 11, 14, 16, 17 and 22), ASD genes (e.g., 15, 16, 17 and 22), and/or obesity genes (e.g., 11, 15, 16, 17 and 22) than predicted based on their chromosome length. However, eight chromosomes (4, 5, 8, 9, 10, 13, 18 and Y) contained proportionally fewer genes representing the three gene groups than expected based upon their size. In addition, 13 chromosomes (2, 3, 4, 5, 6, 7, 8, 9, 10, 13, 18, X and Y) contained fewer protein-coding genes, 11 chromosomes (4, 5, 6, 8, 9, 10, 13, 14, 18, 20 and Y) had fewer ASD genes, and 10 chromosomes (3, 4, 5, 7, 8, 9, 10, 13, 14 and 18) had fewer obesity genes than expected based on size. The X chromosome had the greatest proportion of ASD genes above the expected level, chromosome 19 had the greatest proportion of protein-coding genes above expected, and chromosome 16 had the greatest proportion of obesity genes, again above expected based upon the length of the chromosome.

### 2.2. Chromatin Subtyping by Giemsa Band Intensity and Fragile Sites

#### 2.2.1. Chromatin Subtyping

**Figure 4:**
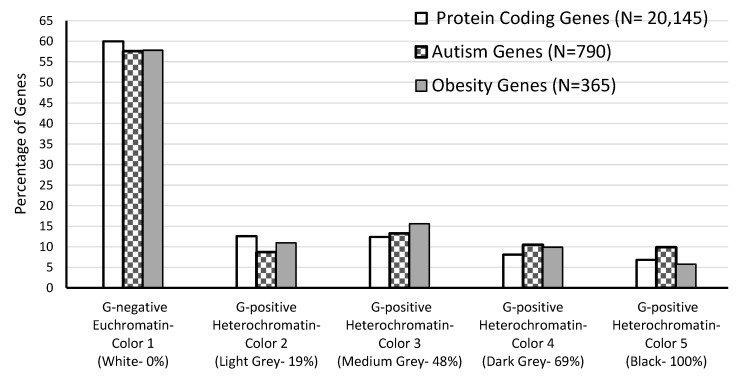
Genome-wide distribution of protein-coding, autism and obesity genes by Giemsa band intensity rating. Calculations excluded genes located in the qh, centromeric and acrocentric chromosome p arm regions.

Analysis of chromatin subtype considered both genome-wide and chromosome-level gene distributions, and, as may be anticipated, the greatest number of genome-wide protein-coding (60%), ASD (57.6%) and obesity (57.8%) genes were located in the G-negative euchromatin band type (see Table 3). No significant differences were found between the proportion of ASD, obesity or protein-coding genes for euchromatin *vs.* overall heterochromatin regions (χ^2^ = 2.4, df = 2, *p* = 0.29). However, an asymmetric distribution pattern of ASD, obesity and protein-coding genes over the range of G-band intensity levels was observed across the genome. Figure 4 shows the proportion of genes per group by chromatin G-band intensity with light to dark banding scaled numerically by color from 1 to 5. As shown, the proportion of protein-coding genes progressively decreased from 12.6% to 6.8% as the banding color intensity increased (became darker representing colors 2 through 5) for the heterochromatin regions. However, the ASD and obesity genes appear to cluster more in the G-positive heterochromatin (colors 2–5) bands, particularly in medium grey (color 3) and dark grey (colors 4 and 5) as compared to light grey (color 2) and G-negative euchromatin (white, color 1) bands. The lowest number of ASD genes (*i.e.*, 69) were found in the G-positive heterochromatin band color 2 and the lowest number of obesity genes were found in the G-positive heterochromatin band color 5 (see Table 3). This overall difference was statistically significant (χ^2^ = 31.6, df = 8, *p* < 0.0001). Examination of standard residuals for ASD genes showed z = +2.21 (G-positive band color 4) and z = +3.14 (G-positive band color 5) relative to obesity and protein-coding genes and z = −2.97 for G-band color 2 (Table 4).

**Figure 5:**
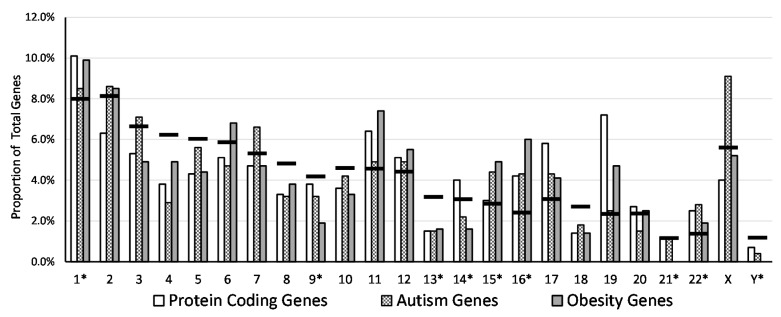
Distribution and proportion of protein-coding, autism and obesity genes by chromosome. * = Length and genes of qh, centromeric and/or acrocentric chromosome p arm regions were excluded. Horizontal black line (—) for each chromosome represents the expected proportion of genes based on chromosome size relative to the total length of all chromosomes summed together.

Further analysis at the chromosome level (see Figure 5) showed that the X chromosome harbored a 2.28-fold higher proportion of ASD genes in relation to protein-coding genes, followed by chromosome 15 with a 1.5-fold higher portion of ASD genes; chromosome 7 (1.4-fold); chromosomes 2 (1.4-fold); chromosome 3 (1.3-fold); and chromosome 5 (1.3-fold). Examination of gene distributions for ASD, obesity and protein-coding genes on the X chromosome did not achieve statistical significance (χ^2^ = 4.1, df = 2, *p* = 0.13). The chromosomes with the highest proportion of ASD genes also had known ASD gene hotspots, such as: 15q11-q13, 15q13.3, 7q11.23, 7q32.2, Xq28, 22q13 [29], indicating these gene hotspots are dispersed throughout the genome. Chromosome 19 contained the lowest portion of ASD genes in relation to the number of protein-coding genes (0.4-fold differences). Unlike the X chromosome with the highest proportion of ASD genes (*i.e.*, 2.3-fold) in relation to protein-coding genes, the Y chromosome contained nearly four-fold fewer ASD genes (*i.e.*, 0.6-fold) in relation to protein-coding genes. Chromosome 15 had the greatest disparity between protein-coding and obesity genes (2.5-fold) followed by chromosome 9 with a twofold difference and chromosome 19 with 1.53-fold. The least difference in the proportion of obesity genes relative to protein-coding genes was seen for chromosome 15 (0.6-fold), while excluding chromosome 21 and Y as they did not contain recognized obesity genes.

To further understand and conceptualize the different gene group distributions across chromosomes, chromosomes were ranked based on length, number of protein-coding, ASD, and obesity genes from the longest length or with the most number of genes to the shortest length or with the least number of genes per each gene group (see Table 5). As previously described, the gene number is generally positively correlated with the chromosome length which is reflected in the rank designation for the different gene groups. Deviations from this pattern were found for chromosome 4 with a low ranking for the number of protein-coding and autism genes compared with a relatively high rank based on length.

Heterochromatin regions are historically associated with gene silencing or inactivity and areas of less genomic activity [16], but the distribution of euchromatin and heterochromatin regions is not uniform across the chromosomes. We found that heterochromatin DNA regions did contribute genetically with 42.4% of the ASD genes and 42.2% of obesity genes located within the G-positive heterochromatin regions (colors 2–5). This observation was similar to the distribution patterns seen in genome-wide protein-coding genes. Furthermore, our results when analyzing the distribution of genes among G-negative euchromatin and G-positive heterochromatin bands at the chromosomal level supported that heterochromatin regions are also places of active gene expression and not silenced, with an average distribution of the three gene groups across each chromosome with 60% for euchromatin bands and 40% for heterochromatin bands (see Table 2). However, the majority of genes were located within the heterochromatin DNA regions in several select chromosomes with chromosomes 6, 11 and 19 containing ≥50% of the protein-coding genes as opposed to euchromatin DNA regions. Similarly, chromosomes 1, 6, 10 and 14 contained ≥50% of the recognized ASD genes in the heterochromatin *vs*. euchromatin regions while chromosomes 8, 10, 13, 19, 22 and X contained ≥50% of the recognized obesity genes in the heterochromatin *vs.* euchromatin regions.

The G-negative *vs.* G-positive chromosome banding is based on Giemsa staining patterns. Giemsa binds to phosphate groups along the chromosome and perhaps more intensely to those phosphate groups at regions of DNA where there are high amounts of adenine-thymine bonding and that are relatively gene poor. In contrast, less condensed chromatin which tends to be rich in guanine and cytosine (GC-rich) and more transcriptionally active incorporates less Giemsa stain. These regions appear as light bands of varying intensities depending on the degree of AT/GC distribution pattern and transcriptional activity within the light banded regions [30,31,32]. The Giesma banding and splitting of bands are evident when reviewing the ISCN (2013) ideograms in progression from the 300, 400, 550, 700, and 850-band levels [18]. Giemsa negative and light band(s) appear *de novo* within Giemsa-positive (dark), and rarely, Giemsa-positive (dark) finer bands have been depicted as appearing from larger Giemsa-negative band regions [33]. Hence, the heterochromatic G-positive bands have been shown to contain long stretches of euchromatin DNA which may become G-negative upon extension of the chromosome length, as observed in prometaphase or late prophase banding patterns which far exceed the high-resolution 850-band level [34]. Conversely, as chromosomes condense during early mitosis, their sub-bands fuse in a highly coordinated fashion [35]. Sub-band fusion occurs when two large sub-bands flanking one minor sub-band come together to form one band, which takes on the cytological characteristics of the original flanking sub-bands, when studied from prophase (>1250 bands per haploid set) to late metaphase (~300 bands). Transcriptionally active autism or obesity-susceptibility genes located within the dark Giemsa bands at the 850-band level may be explained if the chromatin is stretched farther (e.g., >2000 band level at the early prophase stage) as narrow, unrecognized G-negative euchromatin regions are embedded within the larger heterochromatin DNA region [36,37]. Overrepresentation of ASD genes in darker heterochromatin bands may reflect the sequestration of transcriptionally active neurodevelopmental genes to inactive chromosome regions following their phase of functional developmental activity.

#### 2.2.2. Fragile Sites

Chromosome fragile sites are also of interest when examining the location or distribution of protein-coding or disease-causing genes located on chromosome bands within the genome. Fragile sites are highly susceptible to changes, disturbances, and instability and thus might select against the location of important protein-coding genes. Analysis of chromosomal fragile sites, specifically aphidicolin-induced CFSs (aCFSs), has revealed that chromatin band-type coverage was the greatest predictor of genome-wide chromosomal fragility and that the majority of aCFSs were within euchromatin regions [38]. Furthermore, Butler [39] also reported on folate-sensitive fragile sites or lesions located at the 350 (mid-metaphase) chromosome band level from peripheral blood cells in a cohort of 117 males with intellectual disability. All but three chromosomes (*i.e.*, 19, 21 and Y) contained fragile sites in cells grown in folate deficient-culture conditions in Medium 199 [39]. Although chromosome 19 did not show fragile sites, it contained the second highest number of protein-coding genes in our morphometric study. Recent studies have reported fragile sites on chromosome 19, but at a lower number in relationship to other chromosomes with a high number of protein-coding genes [40]. One could speculate that a selection against fragile sites on chromosome 19 could exist and is less likely to be susceptible to chromosome breakage or damage resulting from fragile sites due to the quality and/or quantity of specific genes (e.g., housekeeping) that are important for survival. Links have also been reported between autism and fragile sites (e.g., fragile X syndrome) with fragile site stability involving autism-susceptibility genes impacted by folate levels and metabolism [41]. Folate has a key role in the synthesis of DNA and control of DNA methylation [42].

Additionally, the study by Butler [39] reported that spontaneous fragile sites were more concentrated within G-positive heterochromatin bands as compared to G-negative euchromatin bands (e.g., 157 fragile sites distributed over 171 G-negative euchromatin bands *vs.* 144 fragile sites distributed over 127 G-positive heterochromatin bands excluding centromeric, qh and acrocentric chromosome p arm regions) from 6009 cells grown in folate deficient-culture conditions using Medium 199. There was a lower ratio for the number of euchromatin fragile sites and chromosome bands (*i.e.*, 0.92) as compared to the number of heterochromatin fragile sites and chromosome bands (*i.e.*, 1.13). This difference may suggest that fragile sites tend to appear in areas that are less gene-rich and thus less likely to impact genomic function. The ratio of 1.1 was lower for G-negative euchromatin fragile sites (*N* = 157) and G-positive heterochromatin fragile sites (*N* = 144) compared with the ratio of protein-coding genes at 1.5, ASD genes at 1.4 and obesity genes at 1.4 for G-negative euchromatin and G-positive heterochromatin bands reported in our morphometric study. A better understanding of the effects of autism and obesity-susceptibility genes in relation to location of chromatin type and fragile sites could help researchers in understanding the etiology of autism and obesity, and future studies could analyze the connection between fragile site and gene location and chromatin type for specific gene disorder groups beyond autism and obesity. Furthermore, one could determine if chromatin type has an effect on cancer-susceptibility genes and fragile site location, given the well-documented connection between cancer and fragile sites [43].

The molecular mechanism initiated to silence or activate heterochromatic genes appears to result from a balance between negative factors that promote formation of condensed higher-order chromatin structure and positively acting transcription factors that bind to regulatory sequences which activate gene expression [44]. In general, the acetylation of histones is linked to transcriptional activation with histone acetylation decreasing inter-nucleosome interaction, thereby allowing greater accessibility for gene regulation. Histone methylation of both histones and the DNA molecule further directs gene control implicated in disease which underscores the importance of the functional relationships between histone and DNA methylation in maintaining epigenetic traits. Those ASD and obesity genes that are found to be present in the Giemsa-positive dark regions that are of various shades at the 850-band level are expected to be relatively GC-rich regions in defined euchromatin regions embedded within the current dark Giemsa bands, and transcriptionally active with H3K79me1-active histone modifications, and perhaps, acetylation with H3K27ac [45]. At the fiber FISH chromatin level which is greater than 15-fold magnifications to 850-band ideograms, the so-called heterochromatic—dark band domains contain approximately 17% of active gene expression [37]. Even during the cell division at the metaphase stage, one can expect them to contain brief H3K79me1-rich stretches of nucleosomes/chromatin fiber. Additionally, there are at least 39 histone modifications that are classified into active histone modifications and repressive histone modifications for use in chromatin domain prediction. Active modifications are positively correlated with gene expression levels and are known to mark euchromatin genomic regions, whereas repressive modifications are negatively correlated with expression levels and marking heterochromatic domains. Given the fact that the functionality of protein-coding genes is dynamic (euchromatin to facultative heterochromatin status), and the fact that ASD and obesity-causing genes code for functional proteins—either structural or regulatory proteins—their apparent cytogenetic location at the Giemsa-facultative heterochromatic-dark banded regions of varying intensities, cannot necessarily be construed as entirely indicative of their functional inactivation. Hence, the importance of studying histone modifications is emphasized, as mutations in this process may affect most gene structure and biological processes [46,47].

The current study at the 850-band level shows a threefold decrease in the number of protein-coding genes as well as the ASD and obesity genes with an overrepresentation of ASD genes in the facultative G-positive heterochromatic dark band regions. Our examination of the distribution of the protein-coding genes, autism and obesity genes per chromosome and assessment of the disease gene frequency in relation to the chromosome length and G-band characterization was undertaken to examine for bias or skewness in the distribution of disease genes. It is established that a subset of current human chromosome arms or segments were derived from acrocentric chromosomes of ancestral origin including chromosomes 2 and 4 (with relatively recent changes) [48,49]. In addition, the Y chromosome was recently derived or evolved from the X chromosome through shedding of duplicated genes and by retaining and amplifying male-specific genes to compensate for the loss of recombination in order to maintain the integrity of those genes in the absence of recombination with the X chromosome [50].

## 3. Experimental Section

The individual length of each G-negative euchromatin and G-positive heterochromatin chromosome bands was measured in millimeters and recorded from the 850-band high-resolution ideograms supplied by the International Society of Chromosome Nomenclature (ISCN) 2013 based on scaled cytological data [18] then utilized to calculate the ratio of the two band types per chromosome and chromosome arm. Digital representations were prepared for each chromosome with scaled and stacked images that summarized euchromatin and heterochromatin band distributions over the length of each chromosome (see Figure 2). The images were devoid of centromeric regions, constitutive heterochromatic regions at 1qh, 9qh, 16qh, and Yqh, and acrocentric short (p) arms for chromosomes 13, 14, 15, 21 and 22. To increase size and improve resolution for measurement purposes, each ideogram was uniformly magnified (×125%) from the original source [18]. Each scaled image of the summarized euchromatin and heterochromatin chromosome regions was carefully measured using a battery-operated Pittsburgh 6-inch digital caliper (Harbor Freight Tools, Camarillo, CA, USA) and recorded to the one-hundredth of a millimeter. In addition, the total length of G-negative euchromatin and G-positive heterochromatin bands was measured and recorded for each individual chromosome and summarized over the entire genome. The total length of euchromatin and heterochromatin regions per chromosome was then used to calculate the percent length for each band type by dividing the length of each chromatin region for a given chromosome by the overall length of the whole genome.

The location of known genome-wide protein-coding genes was displayed on electronic high-resolution chromosome ideograms supplied by the Genome Reference Consortium at the public access authoritative Ensembl website (available at: http://uswest.ensembl.org/Homo_sapiens/Location/Genome) via whole-genome location-based displays [21]. The ideograms were last accessed from the website on 7 December 2014 and updated in August of 2014 using Gencode version GENCODE 21. The total number of genome-wide protein-coding genes for each band was estimated based upon the length in millimeters of each histogram bar illustrating the location of protein-coding genes on the images and arranged perpendicularly to the axis of the high-resolution G-banded represented chromosome ideograms [21]. Figure 1 provides an example of the images used and protein-coding gene distribution, along with the distributions and numbers of recognized ASD and obesity gene sets at the chromosome band level. The total length of the measured histogram bars representing the number of protein-coding genes was then summarized for each chromosome. This sum was divided by the number of protein-coding genes for that specific chromosome. The resulting quotient was used to derive the number of protein-coding genes in each individual histogram bar unit representing these genes in humans. Protein-coding genes were then counted by rounding to the nearest number representing a gene. Each horizontal bar was matched with its respective specific band on the chromosome, showing the distribution and location of the genes. If a band had multiple protein-coding gene histogram bars, the sum of all the bars for that band was then calculated to identify the number of genes per high-resolution chromosome band. Because we focused on euchromatin and heterochromatin chromosome regions, the negligible number of protein-coding genes located at the centromeric, qh and acrocentric chromosome short (p) arm regions were excluded from data analysis. The total number of genome-wide protein-coding genes calculated equaled 20,145, in agreement with the total gene count information from the Ensembl website.

The Ensembl 2014 chromosome ideograms matched the ISCN 2013 chromosome ideograms [18,21], except for seven locations. In each of these instances, the Ensembl ideogram did not contain sub-bands as noted in the ISCN ideograms (e.g., the Ensembl 2014 ideogram showed one band at 1q32.1, whereas the ISCN 2013 ideogram showed three sub-bands at 1q32.11, 1q32.12, and 1q32.13). In these instances, the total number of protein-coding genes for the band on the Ensembl 2014 ideogram was divided by three and evenly distributed across the three more specific sub-bands found in the ISCN 2013 ideogram. The fractional number of genes were rounded to the nearest whole number.

The comparison of protein-coding genes was undertaken in the current study with the 792 neurodevelopmental or functional genes currently recognized as playing a role in ASD and their known chromosome locations [27]. Two ASD genes were excluded from analysis because of their location in a qh, centromeric, or acrocentric chromosome p arm region. Locations for the remaining 790 genes were further refined based on their promotor-molecular locations on the chromosome using website sources such as the Online Inheritance of Man (OMIM) (available at: www.omim.org) and GeneCards (available at: https://www.genecards.org). Additionally, the recognized genes for ASD were then identified as either located on the G-negative (light) euchromatin or G-positive (dark) heterochromatin bands represented in the 850-band chromosome ideograms supplied by ISCN. The distribution of genes from a second gene group representing the obesity-related genes with metabolic or systemic function were also evaluated in a similar manner. A list of 365 clinically relevant and candidate genes for obesity were analyzed (five genes were excluded from the master list of 370 reported obesity genes [26]) based on their location in the qh, centromeric, or acrocentric chromosome p arm regions, and their locations were further refined as stated above before being placed on G-negative euchromatin or G-positive heterochromatin bands on each chromosome.

We further investigated differences among the varying levels of G-positive banding intensity (coloring) within specific chromosome regions compared with the single level (white color) for G-negative bands. Adobe Photoshop (2015) (Adobe Systems Incorporated, San Jose, CA, USA) was used to determine the levels of the G-positive band shading intensity (scaled numerically from 2 to 5 for lightest to darkest color) patterns within the heterochromatin regions on the high-resolution Ensembl chromosome ideograms. Each distinct band on the chromosome was scanned and examined using the Color Picker Tool in Adobe Photoshop to determine the degree of color intensity or darkness. Briefly, the tool pointer was hovered over the band and the color recorded using a greyscale format from 0% (white) to 100% (black). There were a total of five different greyscale grades, one for G-negative and four for G-positive bands. White (color 1) represented the G-negative euchromatin band regions, while 19% were light grey (color 2), 48% medium grey (color 3), 69% dark grey (color 4), or 100% black (color 5) representing the G-positive heterochromatin band regions. The short (p) arm of the acrocentric chromosomes (*i.e.*, 13, 14, 15, 21, and 22) and qh regions (*i.e.*, 1, 9, 16, and Y) which lack protein-coding genes were excluded from the analysis. The Chi-Square test was used to compare the distribution of ASD, obesity and protein-coding genes among euchromatin *vs*. heterochromatin regions genome-wide. Due to the known male prevalence of ASD, *ad hoc* analyses also considered the relative distribution of ASD, obesity and protein-coding genes for euchromatin *vs*. heterochromatin regions of the X chromosome alone.

In review of the literature and our research to address gene-chromosome band relationships (location and type), we reviewed published resources pertaining to the chromosome distribution and signal patterns associated with DNA methylation. We previously reported global DNA promoter methylation patterns from the frontal cortex of alcoholics and controls and found the methylation density patterns targeting CpG islands of the promoters of genes correlated with recognized chromosome banding patterns [51]. Higher CpG methylation peaks or intensity readings at genes were found in G-negative (more genes) chromosome bands and decreased size of peaks in the G-positive (fewer genes) bands in alcoholic and control subjects. For example, we found that 16 of the 20 highest methylation peaks representing CpG islands at gene promoters on chromosome 6 were located on G-negative bands when superimposed over the human chromosome 6 ideogram (data not shown). Thus, the results of our methylation signal data based on global DNA promoter methylation found in high-resolution methylation-specific microarrays and characterization in alcoholics were similar to the visual chromosome G-positive and G-negative bands associated with the distribution of protein-coding genes in ideograms.

## 4. Conclusions

Our study supports recent trends implicating genes located in heterochromatin regions as playing a role in biological processes including neurodevelopment and function, specifically genes associated with autism spectrum disorder (ASD). For example, almost one-half of the genome-wide protein-coding genes and genes associated with ASD and obesity were located in the G-positive heterochromatin regions. We found a significant overrepresentation of genes contributing to neurological function or development (*i.e.*, ASD) in darker G-positive heterochromatin bands relative to protein-coding genes and those with a systemic basis of function or disease (*i.e.*, obesity). Some genes were overly represented in specific chromosomes (e.g., X chromosome and ASD genes). One could propose analyzing these cytogenetic regions (individually and collectively) in the future by examining the ratios between the protein-coding and ASD genes to further identify ASD gene congregation (if any) in these known ASD-critical regions (e.g., 15q11-q13, 7q11.23, *etc.*) in the chromosomes represented in ideograms, and to simultaneously check for protein-coding gene status at possibly unstable and highly recombinant chromatin locations. Similar questions could be raised regarding the obesity-related genes and they could be examined for obesity gene congregation on chromosome ideograms. Our observations may stimulate future research to analyze the distribution of other gene groups in relationship to chromatin regions and bands including the examination of epigenetically and bioinformatically defined methylation domains in chromatin from different tissues (e.g., Schroeder *et al.*, 2011 [52]). In addition, of interest to genetic researchers would be to investigate genes found in different cell sources with distinct functions, such as ASD genes expressed in neuron cells and obesity genes in hepatic cells, and their relationship, if any, between the location and position of genes having different functions (*i.e.*, ASD genes on behavior/cognition expressed in the central system or brain and obesity-related genes expressed systemically or in peripheral systems). The study of specific G-band (positive or negative) patterns and respective histone maps may correlate with different genome-wide expression, and accessibility could utilize the data from the recently published Epigenome Roadmaps project (available at: http://www.roadmapepigenomics.org/) and yield new information about clustering of specific groups of genes at the tissue or organ (brain, liver, blood, adipose) level or disease (ASD, obesity) state [53]. The above in-depth analysis is beyond the scope of our descriptive approach of examining the location and interaction of protein-coding, ASD and obesity genes at the chromosome or chromosome ideogram or band level. Our study may help researchers gain a better understanding of the foundation of gene clustering and distributions in relationship to chromosome size and proportion of chromosome banding type, as well as specific gene group distribution with similar or dissimilar function as a hierarchical arrangement of gene function and dynamics.

## Figures and Tables

**Table 1:** High-resolution chromosome ideogram measurements and Giemsa banding patterns.

Chromosome	Number of Bands	Total Length (mm)	% of Total Length of all Chromosomes	Total Euchromatin Length (mm)	% Euchromatin	Total Heterochromatin Length (mm)	% Heterochromatin	Ratio of Euchromatin/Heterochromatin
1 *	62	178.9	8.0	101.0	56.4	78.0	43.6	1.30
2	62	182.0	8.1	106.3	58.4	75.7	41.6	1.40
3	59	148.8	6.6	82.4	55.4	66.4	44.6	1.24
4	45	139.5	6.2	76.5	54.9	62.9	45.1	1.22
5	45	134.9	6.0	82.8	61.4	52.1	38.6	1.59
6	48	131.2	5.9	84.6	64.5	46.6	35.5	1.81
7	42	118.9	5.3	72.6	61.1	46.2	38.9	1.57
8	38	107.9	4.8	67.5	62.5	40.4	37.5	1.67
9 *	38	93.6	4.2	58.0	61.9	35.6	38.1	1.63
10	40	102.9	4.6	65.2	63.4	37.7	36.6	1.73
11	34	102.1	4.6	63.7	62.4	38.4	37.6	1.66
12	39	98.8	4.4	62.3	63.0	36.6	37.0	1.70
13 *	31	71.1	3.2	42.6	59.9	28.6	40.1	1.49
14 *	27	68.6	3.1	41.9	61.1	26.7	38.9	1.57
15 *	27	63.7	2.8	41.2	64.7	22.5	35.3	1.83
16 *	22	54.0	2.4	40.9	75.7	13.1	24.3	3.11
17	22	68.7	3.1	51.6	75.1	17.1	24.9	3.02
18	18	60.5	2.7	39.5	65.3	21.0	34.7	1.88
19	15	52.4	2.3	36.2	69.0	16.3	31.1	2.22
20	18	52.8	2.4	36.6	69.2	16.3	30.8	2.25
21 *	9	25.9	1.2	16.7	64.3	9.3	35.7	1.80
22 *	11	30.8	1.4	25.0	81.1	5.8	18.9	4.28
X	38	125.3	5.6	76.6	61.1	48.7	38.9	1.57
Y *	8	26.4	1.2	17.7	67.0	8.7	33.0	2.03
Total/Average	798	2239.7	100.0	1389.1	56.4	850.7	43.6	1.63

Chromosome bands and lengths were measured from ISCN (2013) high-resolution ideograms magnified ×125%. Measurements do not reflect the actual size of human mitotic metaphase chromosomes. * = Length of qh, centromeric and/or acrocentric chromosome p arm regions were excluded.

**Table 2:** Protein coding gene distribution among G-negative euchromatin and G-positive heterochromatin chromosome regions and relationship to autism and obesity genes.

Chromosome	PCG Sum	% of Total PCG	PCG in Eu	% of PCG in Eu	PCG in Het	% of PCG in Het	AG Sum	% of Total AG	AG in Eu	% of AG in Eu	AG in Het	% of AG in Het	OG Sum	% of Total OG	OG in Eu	% of OG in Eu	OG in Het	% of OG in Het
1 *	2056	10.1	1259	61.2	797	38.8	67	8.5	32	47.8	35	52.2	36	9.9	19	52.8	17	47.2
2	1255	6.3	867	69.1	388	30.9	68	8.6	40	58.8	28	41.2	31	8.5	19	61.3	12	38.7
3	1069	5.3	623	58.3	446	41.7	55	7.1	28	50.9	27	49.1	18	4.9	11	61.1	7	38.9
4	763	3.8	467	61.2	296	38.8	23	2.9	16	69.6	7	30.4	18	4.9	12	66.7	6	33.3
5	864	4.3	513	59.4	351	40.6	44	5.6	29	65.9	15	34.1	16	4.4	12	75.0	4	25.0
6	1041	5.1	520	50.0	521	50.0	36	4.7	13	36.1	23	63.9	25	6.8	13	52.0	12	48.0
7	962	4.7	655	68.1	307	31.9	52	6.6	27	51.9	25	48.1	17	4.7	10	58.8	7	41.2
8	662	3.3	409	61.8	253	38.2	25	3.2	17	68.0	8	32.0	14	3.8	7	50.0	7	50.0
9 *	769	3.8	529	68.8	240	31.2	25	3.2	17	68.0	8	32.0	7	1.9	5	71.4	2	28.6
10	737	3.6	401	54.4	336	45.6	33	4.2	15	45.5	18	54.5	12	3.3	5	41.7	7	58.3
11	1284	6.4	590	46.0	694	54.0	39	4.9	24	61.5	15	38.5	27	7.4	16	59.3	11	40.7
12	1037	5.1	610	58.8	427	41.2	39	4.9	25	64.1	14	35.9	20	5.5	14	70.0	6	30.0
13 *	311	1.5	181	58.2	130	41.8	12	1.5	6	50.0	6	50.0	6	1.6	2	33.3	4	66.7
14 *	807	4.0	550	68.2	257	31.8	17	2.2	8	47.1	9	52.9	6	1.6	4	66.7	2	33.3
15 *	604	3.0	340	56.3	264	43.7	35	4.4	20	57.1	15	42.9	18	4.9	12	66.7	6	33.3
16 *	852	4.2	622	73.0	230	27.0	34	4.3	23	67.6	11	32.4	22	6.0	12	54.5	10	45.5
17	1176	5.8	696	59.2	480	40.8	34	4.3	26	76.5	8	23.5	15	4.1	13	86.7	2	13.3
18	278	1.4	170	61.2	108	38.8	14	1.8	9	64.3	5	35.7	5	1.4	4	80.0	1	20.0
19	1422	7.2	708	49.8	714	50.2	20	2.5	10	50.0	10	50.0	17	4.7	6	35.3	11	64.7
20	539	2.7	337	62.5	202	37.5	12	1.5	6	50.0	6	50.0	9	2.5	5	55.6	4	44.4
21 *	221	1.2	157	71.0	64	29.0	9	1.1	5	55.6	4	44.4	0	0.0	0	0.0	0	0.0
22 *	487	2.5	301	61.8	186	38.2	22	2.8	17	77.3	5	22.7	7	1.9	2	28.6	5	71.4
X	811	4.0	503	62.0	308	38.0	72	9.1	40	55.6	32	44.4	19	5.2	8	42.1	11	57.9
Y *	138	0.7	75	54.3	63	45.7	3	0.4	2	66.7	1	33.3	0	0.0	0	0.0	0	0.0
Total/Average	20,145	100.0	12,083	60.0	8062	40.0	790	100.0	455	57.6	335	42.4	365	100.0	211	57.8	154	42.2

* = Length and genes of qh, centromeric and/or acrocentric chromosome p arm regions were excluded. PCG = Protein Coding Genes, AG = Autism Genes, OG = Obesity Genes, Eu = Euchromatin, Het = Heterochromatin.

**Table 3:** Summary data of chromosome bands and genes by group for each chromatin type.

Chromatin Type	Number of Bands	PCG Sum	% of Total PCG	AG Sum	% of Total AG	OG Sum	% of Total OG
G-negative Euchromatin (Color 1)	417	12,083	60.0	455	57.6	211	57.8
G-positive Heterochromatin (Colors 2–5)	381	8062	40.0	335	42.4	154	42.2
*Heterochromatin-Color 2*	*89*	*2547*	*12.6*	*69*	*8.7*	*40*	*11.0*
*Heterochromatin-Color 3*	*123*	*2499*	*12.4*	*105*	*13.3*	*57*	*15.6*
*Heterochromatin-Color 4*	*88*	*1638*	*8.1*	*83*	*10.5*	*36*	*9.9*
*Heterochromatin-Color 5*	*81*	*1378*	*6.8*	*78*	*9.9*	*21*	*5.8*
Total	798	20,145	100.0	790	100.0	365	100.0

PCG = Protein-coding Genes, AG = Autism Genes, OG = Obesity Genes. Number of bands and genes were calculated after removal of qh, centromeric and acrocentric chromosome p arm regions.

**Table 4:** Summary of the percentage deviation with standardized residuals (z-scores) for chromosome bands and genes by group and chromatin type.

Group	G-Negative Euchromatin Band		G-Positive Heterochromatin Bands							
	*Color 1*		*Color 2*		*Color 3*		*Color 4*		*Color 5*	
	*Number of Genes*	*Percentage (z-Score)*	*Number of Genes*	*Percentage (z-Score)*	*Number of Genes*	*Percentage (z-Score)*	*Number of Genes*	*Percentage (z-Score)*	*Number of Genes*	*Percentage (z-Score)*
ASD	455	−3.8 (−0.82)	69	−30 (−2.97) *	105	+6.4 (+0.63)	83	+27.4 (+2.21) *	78	+42.4 (+3.14) *
Obesity	211	−3.4 (−0.51)	40	−12.1 (−0.82)	57	+25 (+1.69)	36	+19.6 (+1.07)	21	−17 (−0.86)
PCG	12,083	+0.2 (+0.23)	2547	+1.4 (+0.7)	2499	−0.7 (−0.35)	1638	−1.4 (−0.58)	1378	−1.4 (−0.51)

Chi-Square test percentage deviation and standardized residuals for each cell. ASD = Autism spectrum disorder, N = 790 genes; Obesity, N = 365 genes; PCG = Protein-coding genes, N = 20,145 genes. * Greater than or less than 2 standard deviational z-scores.

**Table 5:** Chromosome rank order by length and gene frequency distribution for protein coding, autism and obesity genes.

Chromosome	Length Rank	Ratio of Euchromatin/Heterochromatin	PCG Number Rank	AG Number Rank	OG Number Rank
1 *	2	1.30	1	3	1
2	1	1.40	4	2	2
3	3	1.24	6	4	8
4	4	1.22	15	16	9
5	5	1.59	10	6	13
6	6	1.81	7	9	4
7	8	1.57	9	5	11
8	9	1.67	12	14	15
9 *	13	1.63	14	15	18
10	10	1.73	16	13	16
11	11	1.66	3	7	3
12	12	1.70	8	8	6
13 *	14	1.49	21	21	20
14 *	15	1.57	13	19	21
15 *	17	1.83	18	10	10
16 *	19	3.11	11	11	5
17	16	3.02	5	12	14
18	18	1.88	22	20	22
19	21	2.22	2	18	12
20	20	2.25	19	22	17
21 *	23	1.80	23	23	23
22 *	22	4.28	20	17	19
X	7	1.57	12	1	7
Y *	24	2.03	24	24	24

Chromosomes were ranked from 1 (greatest) to 24 (least) based on either length or number of genes per chromosome. PCG = Protein Coding Genes, AG = Autism Genes, OG = Obesity Genes. * = Length and genes of qh, centromeric and/or acrocentric chromosome p arm regions were excluded.

## Abbreviations

ASD	Autism Spectrum Disorder
AT	Adenine-Thymine
CFS	Chromosome Fragile Sites
df	Degrees of Freedom
DNA	Deoxyribonucleic Acid
GC	Guanine-Cytosine
ISCN	International Society of Chromosome Nomenclature
OMIM	Online Inheritance of Man
